# A Week in the Life: Pediatric Palliative Care through the Eyes of a Medical Student

**DOI:** 10.3390/children8110971

**Published:** 2021-10-27

**Authors:** Eileen Williams, Jill Ann Jarrell, Jared Rubenstein

**Affiliations:** 1 Baylor College of Medicine, Houston, TX 77030, USA; eileenw@bcm.edu (E.W.); jajarrel@texaschildrens.org (J.A.J.); 2 Division of Palliative Care, Department of Pediatrics, Texas Children’s Hospital, Houston, TX 77030, USA

**Keywords:** narrative medicine, reflection, pediatric palliative care, medical education

## Abstract

To complete the curriculum, learners rotating through a pediatric palliative care service are asked to submit a piece of reflective writing. Here, we share an edited version of the narrative one student submitted, accompanied by a brief consideration of the numerous benefits of reflective writing for medical trainees (including improved communication and professionalism skills, as well as increased levels of empathy and comfort when facing complex or difficult situations). Additionally, we describe how brief personal narratives may serve to reduce common misconceptions and confusion by educating patients, families, and clinicians about the reality and the role of pediatric palliative care.

## 1. Introduction

As a palliative care team at a teaching hospital, we ask all the learners that rotate with our team to write a reflection piece at the end of the rotation. While the majority of trainees write a piece at the end as a synthesis of the experience, one medical student (one of this paper’s authors, E.W.) kept a daily journal of her experience. The outcome was a remarkable piece that felt like a narrative “week in the life” of a pediatric palliative care team. We use an edited version here (with patient and clinician details changed for anonymity) to discuss the importance of reflective writing in pediatric palliative care education. Additionally, we propose that brief narratives of this form can serve a crucial role in educating others about pediatric palliative care, both patients and families as well as clinicians in other fields.

## 2. Monday

Day one of palliative care, and we very much hit the ground running. First, I met the clerkship director who explained how the service runs. He then introduced me to the rest of the team, whose members ranged from physicians and nurses to social workers and chaplains. Previously, my most “interdisciplinary” experience in medical school was a required “team-building” course with PA students, during which we were forced to construct dry spaghetti towers. It was not exactly a meaningful experience. On palliative care, I was struck by the true cohesion of the team, how easily everyone moved in the space. I watched a physician joking with a chaplain, and I knew I was in a different world.

Sign-out was a rapid-fire review of all the patients on service, but before we could even start, our clinical pharmacist tapped my arm from across the conference table and flashed a sheet of paper in my direction with the following written on it: WOLST and a room number. Confused, I scanned through my mental preclinical dictionary of medical terminology, drawing a blank. We certainly never learned this acronym in class, but I managed to deduce “withdrawal of life-sustaining treatments”. It would be the first, but certainly not the last, time I encountered a question not covered in the classroom during a lecture. I learned that (despite common misconceptions) a WOLST is entirely different than physician aid-in-dying or euthanasia. We never give medication with the goal to end a person’s life, only to keep the patient comfortable and avoid unnecessary suffering.

We spent most of the morning rounding and prepared for our 2 p.m. meeting with this patient’s parents. If we did proceed with the WOLST, we still had many details to decide, including: Which medications should we continue? How do we draw a line between managing symptoms and treating disease? Could we give appropriate dosing without needing innumerable tubes and wires? Before this rotation, end-of-life conversations had always centered on one question, i.e., Should we withdraw life-sustaining treatment? For the first time, I realized that the answer is not actually a simple yes/no. I was quickly learning how complex medical management and technical expertise are both crucial to palliative care.

In the afternoon, we spoke to the whole treatment team, i.e., neurology, intensive care, surgery, and cardiology. Before our family meeting, it was essential that the team be on one page. We reviewed what we knew, i.e., this infant had suffered an objectively large (subjectively devastating) neurologic injury and would never achieve normal development. What struck me were all the lingering uncertainties. We could not determine how long the patient would survive after extubation, if he would breathe on his own, when his heart might slow to a fatal rate. We could withdraw life-sustaining therapies, and the patient could still live. I had never considered that possibility. I could not imagine experiencing the immense anticipatory grief of a child’s death, only to have to entirely reimagine life in limbo.

After our discussion, we filed into a large conference room. Looking around the crowded table, I imagined how terrifying a scene it must be for a parent. Our patient was only three weeks old, but already had more subspecialists than many adults see in a lifetime. Fortunately (as much as anything can be fortunate in this situation), the parents were decisive and loving. Recognizing the minuscule chance for long-term recovery with an acceptable quality of life for them, they decided to proceed with the WOLST. As the patient’s father put it, “We want to do things for him, not to him.” With the parents and team in agreement, the decision was indeed made to extubate and redirect to comfort care.

Later that day, when life-sustaining treatments were discontinued, the patient died relatively quickly and peacefully. Nevertheless, a million “what if” scenarios were ringing in my ears. What if the parents did not agree with the medical team? Or, what if we had extubated, and our predictions were wrong? What if he had lived? At every possible branching point, a million different complications could arise. No matter what we do, I am reminded that medicine is largely defined by uncertainty. As providers, we must remain humble.

## 3. Tuesday

During my first rotation (surgery), students arrived at the hospital by 4:45 a.m. We had to finish rounding early and be ready for the OR by 7 a.m. In practice, this time crunch meant that we ran from room to room, poking a head through the doorway and perhaps palpating a patient’s abdomen. More often than not, the patient and family were still asleep. We either risked the wrath of our poor, exhausted patients, or did everything in our power not to wake them up.

Rounding with palliative care was a different experience entirely. We waited until at least 9 a.m., and if a patient or parent was still sleeping, we came back later. Our goal was not merely another checkmark on a checklist, but to support families and patients. We did not bring news, but we did help process it.

Today, we visited an infant who had presented initially with agitation, fever, tachycardia, and septic shock, dysregulated, screaming, and arching his back in distress. Now, after innumerable medications and procedures, he was sleeping in his crib as peacefully as I have ever seen. His face was relaxed, his muscles loose, his breathing easy. We chatted briefly about symptom management and medication, but mostly, we sat with the family. I knew this child still had a long road ahead. It remained impossible to foresee an ultimate outcome, but in the contentment on his face, I could see the purpose of all our interventions. In that moment, I was simply happy that he was alive and loved—regardless of what the past had entailed or what the future would bring.

## 4. Wednesday

On Wednesday, our team had a phone conversation with the mother of a 3-month-old boy. Her poor cell connection made communication difficult, but she managed to convey her message. She did not want the patient, her son, to suffer any longer. He had been in a continuous cycle of slight improvement, and then significant deterioration. She was ready for WOLST.

Initially, palliative care had been consulted because the primary medical team was concerned that his mother did not understand the severity of the situation, the likelihood that her son may not survive. Two weeks ago, her decision would have been perfectly acceptable to, even welcomed by, the team. Now, however, the situation had changed. Our patient was doing slightly better. His pressors were slightly lower, he was showing some respiratory drive, and his copious pleural effusion had decreased somewhat. Given these improvements, the medical team wanted to pursue maximal therapy. Immediately on hearing the mother’s desire to WOLST, another team member suggested an ethics consult, introducing the possibility of trying to oppose this young mother’s clearly stated decision.

While the child’s mother focused on the bigger picture, the primary medical team focused on the details, i.e., the milrinone dosage and the number of milliliters draining from his chest. Of course, I believe that these data points are important; we cannot take care of patients without them. But they also are not the whole story. Overall, this baby remained critically ill and unlikely to survive in any circumstance. Perspective was everything.

## 5. Thursday

After Wednesday’s phone call, the boy’s mother agreed to come into the hospital to proceed with extubation, which was no small feat considering she was a single working mother, without reliable transportation. When she arrived, our team sat firmly in her corner. Yet, the conversation still veered off course. Rather than decide specific details, the mother was forced to rehash the difficult and painful decision with which she had been struggling for so long. Even after yesterday afternoon’s conversation, one specialist advocated for another pleurodesis (which would be the patient’s third), a painful procedure that may or may not help the infected fluid collection around his lungs. He felt he had invested too much to “give up” now. Be that as it may, I could not help but think, this child is not your child. Moral distress is real, and I can see how difficult it would be to withdraw life-sustaining therapy from an infant you had put your entire heart into, particularly if that infant appeared to be getting better. Nevertheless, I do not think it fair that this child’s mother was ultimately pressured to defend and explain her choice—again.

At first, some members of the team thought that this mother was being too optimistic; however, now, she was not being optimistic enough. I found myself frustrated on her behalf, thinking, “What do you want from me?” Even though she had made her final decision, being asked over and over “Are you sure?” by the medical team could not have made things any easier. I truly hope that she does not carry extra guilt because of what we, as a system, did. What we suggest as providers, both explicitly and in our line of questioning, matters to patients.

I am thankful she remained steadfast, but know the meeting could also have gone a different way. In the end, the team agreed to the WOLST, but not that day. She left the hospital with no date set, just a morass of considerations floating through her mind—not the peace and relief I believe she deserved.

## 6. Friday

“How depressing” is the typical response I receive when I tell anyone that I am on a pediatric palliative care rotation. Invariably, when I say that I love it, I am met with a suspicious glance that seems to beg the question, “Are you a total psychopath?”, which is a fair question. No doubt it is hard work but it also makes a difference. In a tangible way, I feel I am helping patients and families, fighting and advocating for them. I try to explain that the bad things already exist, i.e., they are already happening. However, by being there, I get to make them just a tiny little bit less bad, and, despite all the pain, we are never alone to carry this burden.

Every week, we have an interdisciplinary team meeting. We start with a beautiful reading before reflecting on the week’s progress and discussing patients’ next steps. In my experience, palliative care remains unique in the degree to which all team members are supported and encouraged to process difficult emotions on a regular basis. Weekly team meetings are part of fostering this culture, providing a dedicated space for everyone to come together and reflect. As a student, I often feel out of place on the wards, not knowing where I am supposed to be or what I am supposed to be doing. It is easy to bumble through, constantly concerned you are in the way. However, on this rotation, I have truly felt a part of the team. Even if I am not entering orders or calling consults constantly, I know I am participating in patient care. Every single person has welcomed my perspective (despite my fear that it is not entirely legitimate).

For lunch, we ordered from a local restaurant. Fully vaccinated, we celebrated scientific progress against COVID-19 and gathered together in the conference room to eat (at an appropriate physical distance of course). Usually, I never take a “lunch break.” It has always seemed like a silly concept to me. I pack some food and I eat at my desk, multitasking! However, after an intense week, it felt good to take a break, to socialize and laugh, to talk to all the wonderful people around me. Although we could no longer give a hug or squeeze a hand, we found different ways to support each other.

Wrapping up for the week, I felt tired but fulfilled. I sincerely hope that palliative care becomes more integrated into medical education. I know I will carry this experience and perspective through the rest of my career.

## 7. Comments

Reflection has become an important tool to enhance learning in medical education. In particular, narrative medicine as a means of reflection has gained popularity, both in verbal and in written form [1]. Written narrative reflections can be structured, such as responding to prompts, or open-ended, as exemplified by the daily “diary” of E.W.

The utility of reflection in medical education is well-documented. A recent meta-analysis suggests that reflective exercises may increase resident and fellow learning, communication and empathy, professionalism, comfort with complex or difficult situations, and that the act of reflection itself may deepen with subsequent iterations [2]. The benefits of reflective exercises have also been demonstrated among medical students [3].

Written reflections by medical students after a palliative care rotation have demonstrated learnings in medical knowledge and patient care, such as pain and symptom management and assessing patient and family coping; communication; systems-based practice, such as understanding the patient experience; and personal and professional development, such as understanding the mission of medicine and personal growth [4,5,6]. Other studies [7] have shown that narrative reflections by medical students after a palliative care rotation can assess learnings in the domain of professionalism, specifically, the subdomains of self-awareness of one’s own perspectives and biases, and demonstration of empathy and respect.

To the best of our knowledge, there is nothing published on the utility or impact of a narrative reflection by a medical student after a pediatric palliative care rotation. Our attempt at incorporating such an exercise into a pediatric palliative care curriculum is novel and worthy of discussion.

In addition to the educational benefits of reflective writing for the writer, we propose that reflective narratives, specifically of this “day in the life” type, have powerful educational benefits for the reader. Pediatric palliative care continues to be poorly understood. Among children with cancer and their families, the majority report having never heard the term “palliative care” [8]. Additionally, there remains an incomplete understanding of pediatric palliative care among pediatric subspecialists, often accompanied by stigma [9]. Furthermore, when there are negative perceptions about palliative care from patients and families, it has been shown that they may originate from interactions with other healthcare professionals [10].

Much work has been done to improve knowledge of palliative care such as the clear, concise definition created by the Center to Advance Palliative Care (CAPC) https://www.capc.org/about/palliative-care/ (accessed on 22 October 2021). Definitions and platforms like these are helpful, but may not convey the intricacies of palliative care to other clinicians. Most clinicians do not have the time or resources to pursue subspecialized education in palliative care via a formal or informal training such as fellowship or short courses in pain management or communication.

We propose that brief, rich narratives such as this could fill an educational void. Within E.W.’s engaging narrative are descriptions of pain and symptom management, eliciting goals of care, advance care planning, family and staff support, and end-of-life care. All these pediatric palliative care skills are brought to life through narrative with case examples. While not all pediatric clinicians will have the time (or interest) to spend a week with a palliative care team, we believe that reading a narrative such as this may be the next best thing.

## 8. Conclusions

In summary, we believe that reflective narrative writing has an important place in pediatric palliative care education. For the writer, it forms a means to reflect and consolidate lessons learned. For the reader, it provides a lens through which one can gain a rich perspective with concrete examples on the benefits and importance of pediatric palliative care.
